# Prevalence and correlates of psychological distress in a large and diverse public sector workforce: baseline results from Partnering Healthy@Work

**DOI:** 10.1186/1471-2458-14-125

**Published:** 2014-02-06

**Authors:** Lisa Jarman, Angela Martin, Alison Venn, Petr Otahal, Roscoe Taylor, Brook Teale, Kristy Sanderson

**Affiliations:** 1 Menzies Research Institute Tasmania, University of Tasmania, Hobart, Tasmania, Australia; 2 Tasmanian School of Business and Economics, University of Tasmania, Hobart, Tasmania, Australia; 3 Department of Health and Human Services, Tasmanian State Government, Hobart, Tasmania, Australia; 4 Department of Premier and Cabinet, Tasmanian State Government, Hobart, Tasmania, Australia

**Keywords:** Prevalence, Correlates, Workplace, K10, Public sector, Health risk appraisal, Psychological distress

## Abstract

**Background:**

Depressive and anxiety disorders are common among working adults and costly to employers and individuals. Mental health screening is often an important initial strategy, but the resultant data are often of unknown representativeness and difficult to interpret. In a public sector workforce, this study used a brief screener for depression/anxiety to: a) compare prevalence of high psychological distress obtained from a researcher survey with an employer survey and population norms and b) verify whether expected correlates were observed in a screening setting.

**Methods:**

Participants were public servants working for an Australian state government. High psychological distress (Kessler-10 ≥22) stratified by age and sex was compared for a random weighted sample researcher survey (n = 3406) and an anonymous volunteer employer survey (n = 7715). Prevalence ratios (PR) were estimated from log binomial regression.

**Results:**

Referencing the researcher survey, prevalence of high psychological distress was greater by age and sex in the employer survey but was only dependably higher for men when compared with population norms. Modelling suggested this may be due to work stress (effort-reward imbalance) (PR = 3.19, 95% CI 1.45-7.01) and casual/fixed-term employment (PR 2.64, 95% CI 1.26-5.56).

**Conclusions:**

Depression and anxiety screening using typical employer survey methods could overestimate prevalence but expected correlates are observed in a screening setting. Guidance for employers on screening and interpretation should be provided to encourage engagement with mental health prevention and treatment programs in the workplace.

## Background

The common mental disorders of depression and anxiety are among the greatest public health challenges of this era [1]. The earlier issues such as workplace depression are accurately identified and treated, the sooner improvements in work outcomes are likely to occur [2]. As a result, understanding the nature of workplace-based risks associated with poor mental health and addressing these risk factors has been an area of considerable research and practice interest. Researchers have argued that employers should become involved in workforce screening of mental health because the data obtained can assist the development of relevant workplace interventions [3].

Despite the need for workplace action on issues such as depression, transfer of mental health screening into the hands of an employer has specific barriers and challenges [4]. First, employers are typically interested in feedback systems that enable sound management decisions. However, employer surveys are usually anonymous and have volunteer samples [5] that can affect the validity and generalizability of findings.

Second, mental health screening using Health Risk Appraisals (HRAs) has evolved from individually focused, clinical settings [5] rather than work environments. Although there is some evidence supporting the use of HRAs in physical health promotion at work [6] there seems to be a gap in published information on the results obtained by employers who use mental health or associated measures. For example, despite the efforts that Great Britain’s Health and Safety Executive (HSE) has made to develop a viable measure of psychosocial safety, a recent study reported that employers were not using the HSE Indicator Tool as recommended (i.e. used abridged versions, applied only once, substituted with other measures) [7].

Third, employers are realistically concerned about the legal implications of identifying mental health conditions in the workplace [8]. Therefore, to encourage engagement in mental health screening researchers have a role in educating employers about how to interpret findings in view of: a) the limitations of the screening methods and measures used: and b) the expected ranges given the demographic profile of their workforce.

A key population of interest for mental health screening is the public sector (also referred to as the state or government sector), whose employees appear to be vulnerable to poor mental health [3,9]. Given the large number of employees impacted internationally, accurate monitoring of the mental health of public sector employees seems to be an important consideration for public health.

The mental health status of public sector workforces has been researched for several decades (e.g. Whitehall [10], WORC [3]) and has coincided with the implementation of New Public Management (NPM) organisational concepts within civil services around the world. These reforms have led to a wide range of changes to the traditional roles of government and its associated management structures. Reforms have typically included the introduction of private sector concepts such as outsourcing, rationalization, decentralization and performance orientation [11].

There has been considerable debate as to whether organisational change has a negative impact on these employees [12], but evidence from public service populations has reported increased job strain [13], increased presenteeism [14] and sick leave [15], and decreased organisational commitment [13]. Public sector employees have been found to report higher levels of psychological distress than their private sector counterparts [16,17]. However, we do not know if these purported higher levels are a consequence of the different survey methods used, or are due to actual differences in distress in these working populations. In the absence of clarity on these issues, interpretation of whether a public sector result is ‘high’ or not can be furthered by making comparison with working population norms for psychological distress. We acknowledge that population norms can underestimate the prevalence of poor mental health due to non-response bias [18] and as a result comparisons are imperfect. Despite their limitations, these norms can act as a third ‘best estimate’ of prevalence where organisational data are contradictory.

One validated way of assessing risk for depressive and anxiety disorders is the Kessler 10 (K10) brief screening scale [19]. This 10-item scale measures ‘psychological distress’ and has acceptable performance as a screener for DSM-IV depressive and anxiety disorders [20]. Although the K10 can be used to assess the prevalence of psychological distress in workers, it gives no clues about modifiable risk factors influencing poor mental health. When the K10 is applied in a HRA context, it is important to assess whether high distress is associated with the typical demographic, socio-economic, health, and work correlates found in other literature [10]. If the K10 correlates with expected characteristics this result adds evidence of validity to the application of the K10 for detecting high distress in public sector employees.

In this study, results obtained from random weighted and anonymous volunteer HRA surveys using the K10 were compared within a large and diverse public sector organisation. We wanted to assess whether the prevalence of high psychological distress was greater than that of working population norms and verify that the K10 results were associated with expected correlates in a screening setting.

Using a researcher survey as the reference, the aims of this study were to: a) compare the self-reported prevalence of psychological distress measured with a brief screener with that of an employer survey; b) determine whether prevalence differed to normative population data; and c) investigate and classify the socio-demographic, health and work correlates of self-reported high psychological distress.

## Method

### Setting

This study was based in Tasmania, which is an island State of Australia with a population of approximately 500,000 people. The setting for this research was in the form of a partnership, ‘Partnering Healthy@Work’ (PH@W), between the Tasmanian Government and the University of Tasmania. The Tasmanian State Service (TSS) is one of the region’s largest employers with approximately 30,000 employees and incorporates a wide range of government departments (e.g. health, education, environment), occupations and locations (city-based, rural, remote). Since 2009, the Tasmanian Government has invested more than $2 million in workplace health promotion, the “Healthy@Work” (H@W) initiative, targeting their whole workforce. Ethics approval for the study was obtained from the Human Research Ethics Committee (Tasmania) Network (ID: H0010501).

### Study design

Partnering Healthy@Work is a longitudinal evaluation of H@W that includes collection and assembly of data from a range of data sources: a repeated, randomly-selected cross-sectional health workforce survey initiated by researchers (PH@W 2010 and 2013); an anonymous online employer-initiated workforce health survey (H@W 2009 and 2011); and human resource administrative data. This study used data from the first employer (H@W 2009) and researcher (PH@W 2010) workforce surveys, comparing it to normative prevalence data for the Australian and Tasmanian working populations.

### Public sector workforce data sources

The researcher survey was distributed in February 2010 to TSS employees. We selected a 40% random population sample from the total pool of employees, stratified according to employment condition, employment category and agency. The response rate was 28% (N = 3406).

Survey responses were merged with an extract of administrative data from the TSS human resources database to permit analyses according to the key demographic variables of age, sex, employment condition (permanent, fixed-term or casual), employment category (full-time, part-time), annual salary, job classification ((Bands 1-3 [low], Bands 4-6 [mid], Bands 7-8 [high/ manager], Bands 9-10 [very high/ senior executive]), industrial award (blue collar, white collar, service, professional, manager), tenure (within the TSS, by government department) and public sector agency.

Standard survey weighting for the researcher survey was not possible due to very low response rates and zero cells in several strata. Therefore, to adjust for possible response bias, we applied the inverse probability of response weighting method described by Hofler and colleagues [21] based on a model including age, sex, government department, employment category, employment condition, and tenure using the human resources database as the reference population.

The employer survey was made available in an intranet-based format in 2009 to all TSS employees. It was designed by the TSS using a range of pre-existing measures and had a response rate of 25% (N = 7715). This survey asked about age, sex, psychological distress (K10) and a range of lifestyle factors and behaviours that increase the chance of developing chronic health conditions like cardiovascular disease and diabetes. Distinct from the researcher survey, it did not: a) measure broader socio-demographic information, work contextual factors or psychosocial variables; b) use random selection or population stratification procedures; and c) it was anonymous so responses could not be matched to human resources data.

### Comparative normative data sources

We collated population normative data for workers with the same K10 measure of psychological distress from two Australian cross-sectional datasets: a national mental disorder prevalence survey, the National Survey of Mental Health and Well-Being (NSMHWB) [22], and the Tasmanian Population Health Survey 2009 (TPHS) [23] (Table 1). The NSMHWB had a 60% response rate (N =7715 workers). Weighted survey respondent characteristics indicated 50% were female, 57% were married/Defacto and 37% were in the 35 to 54 years age-range. The TPHS had a 70% response rate (N = 3160 workers) and weighted characteristics showed 57% were female, 71% were married/ Defacto and 56% were in the 35-54 years age range. Data were extracted by age, sex, employment status (i.e. workers) and psychological distress.

**Table 1:** Data sources to compare employee psychological distress (Kessler 10)

**Survey**	**Type**	**Population**	**Participation rate**	**n**	**Method**
PH@W 2010^1^	Researcher-initiated	TSS^5^ employees	28%	3406	Random weighted paper and pencil
H@W 2009^2^	Employer-initiated	TSS employees	25%	7715	Non-random anonymous online
TPHS 2009^3^	Population normative	Tasmanian working adults	70%	3160	Random weighted telephone
NSMHWB 2007^4^	Population normative	Australian working adults	60%	5499	Random weighted face-to-face

^1^Partnering Healthy@Work.

^2^Healthy@Work.

^3^Tasmanian Population Health Survey.

^4^National Survey of Mental Health and Well-Being.

^5^Tasmanian State Service.

### Measures

All of the surveys sought information on employed individuals’ age and sex as well as psychological distress using the Kessler-10 (K10) screening scale which scores in a range of 10 to 50 [19,20]. The K10 total psychological distress score was categorized as low (10-21) versus high (22-50) [24]. A high (≥22) or very high score (>29) gives a strong indication of a clinically diagnosable mental health condition [19].

The researcher survey measures, which were used to establish the correlates of psychological distress, included marital status (married or Defacto, not married); education level (up to year 10, up to year 12, post school); general physical health as measured by the SF-12 physical component summary scale [25]; smoking habits (daily smoker, all others) [26]; fruit and vegetable intake (inadequate, adequate) [27]; height and weight to measure body mass index (BMI); alcohol intake [28] (high risk, low risk) measured using national guidelines [29]; and total physical activity (minutes per week) derived from the Long International Physical Activity Questionnaire (IPAQ) [30].

Preferred work hours and contractual work characteristics included days worked (Monday to Friday, days vary weekly, other), schedule worked (regular days, other), and hours worked. Other psychosocial factors were measured using the Effort Reward Imbalance (ERI) questionnaire, which is a commonly applied validated self-report survey with 6 items measuring Effort and 11 items dedicated to Reward. A ratio is calculated for every person by first adding all scores for each of the effort (*e*) and reward (*r*) scales, then applying the formula *e/(r* x *c)* where *c* equals the proportion 6/11 [31]. ERI ratio scores ≥1 are argued to indicate an imbalance of high effort and low reward conditions.

### Statistical analysis

We first derived the prevalence of high psychological distress as proportions of participants with K10 scores of 22 or greater by age and sex in the researcher and employer surveys and the two population normative datasets, using the researcher survey as the reference to calculate variance estimates. The differences between proportions were assessed by calculating standard errors using the standard normal approximation for large samples, and assumed independent sampling. Analysis was stratified by sex. This approach was taken on theoretical grounds [32,33] because of known differences between men and women in correlates of psychological distress.

Second, we used the researcher survey data for multivariable model building [34] with dichotomized psychological distress as the outcome variable. We conducted a univariable analysis, and selected those variables with a p-value less than 0.25 for further analysis. Then we performed logistic regression analyses, entering the selected variables one at a time and conducting a Wald test upon each new variable’s entry to determine if that variable significantly (p < 0.05) increased model discrimination. This process selected the set of variables that provided the best discrimination between low and high psychological distress. ROC values of greater than 0.7 and less than 0.8 model ‘acceptable discrimination’ [34].

Separated by sex, individual covariates were entered one at a time in order of demographic (age, marital status), socio-economic (annual salary, education, occupation), contractual work characteristics (employment condition, employment category, tenure, hours worked, days worked, job classification, schedule worked), health behaviours (general physical health, inadequate fruit and vegetable intake, daily smoker, high risk alcohol, BMI, total physical activity) and psychosocial work environment (preferred hours, ERI). These classification processes did not permit data weighting procedures, so when these analyses were complete, the final sets of variables were used to build log binomial regression models by sex of the predictors of high psychological distress for estimation of weighted prevalence ratios (PR) [35]. All analyses were conducted using STATA 12.1 (StataCorp LP, Texas, USA).

## Results

### Prevalence of high psychological distress

The prevalence estimates of high psychological distress in the two Tasmanian State Service (TSS) workforce surveys are shown in Table 2, stratified by age and sex. The results show that the employer survey estimates of prevalence of psychological distress for were statistically different (*p* < 0.001) to those of the researcher survey for both men (5.9% higher) and women (6.4% higher).

**Table 2:** **Prevalence (%) of high psychological distress**^**1 **^**by age and sex reported in surveys of Tasmanian and Australian employees**

	**Men%(N**^**2**^**)**				**Women%(N)**			
***Age group (years)***	**PH@W 2010**^**3**^	**H@W 2009**^**4**^	**TPHS 2009**^**5**^	**NSMHWB 2007**^**6**^	**PH@W 2010**	**H@W 2009**	**TPHS 2009**	**NSMHWB 2007**
16 - 24	18.7 (16)	23.7 (131)	7.1 (84)	6.2 (512)	16.7 (64)	23.7 (329)	11.1 (80)	11.7 (564)
25 - 34	7.4 (103)	17.3 (394)	6.7 (177)	9.1 (467)	16.4 (337)	22.7 (1001)	9.8 (256)	14.8 (544)
35 - 44	11.8 (222)	17.0 (695)	7.4 (323)	6.5 (687)	13.2 (551)	18.1 (1329)	11.4 (471)	10.7 (674)
45 - 54	11.5 (373)	15.6 (801)	7.8 (392)	8.8 (483)	10.3 (956)	16.1 (1871)	10.1 (586)	10.8 (555)
55 - 64	7.1 (219)	12.0 (417)	7.5 (278)	4.2 (396)	6.7 (477)	14.8 (856)	10.1 (353)	7.7 (379)
65+	0.0 (15)	20.0 (25)	2.5 (80)	3.4 (153)	3.8 (27)	11.4 (35)	9.4 (52)	7.6 (85)
Total	10.2 (948)	16.1 (2463)	7.1 (1334)	6.8 (2698)	11.6 (2412)	18.0 (5421)	10.4 (1798)	11.2 (2801)
*p*^*7*^	ref	<0.001	0.013	0.002	ref	<0.001	0.217	0.651

^1^Prevalence was measured using the K10 and dichotomized as ‘high’ versus ‘low’ distress with K10 scores ≥22 being rated as ‘high’.

^2^N, total number of respondents by category.

^3^Partnering Healthy@Work 2010, researcher-initiated survey of Tasmanian State Service employees (weighted).

^4^Healthy@Work survey 2009, employer-initiated survey of Tasmanian State Service employees (unweighted).

^5^Tasmanian Population Health Survey 2009, population normative data of Tasmanian workers (weighted).

^6^National Survey of Mental Health and Well-Being 2007, population normative data of Australian workers (weighted).

^7^p-value for the differences between total prevalence of psychological distress in the survey, relative to PH@W 2010.

Using the same reference, high psychological distress totals were lower for male Tasmanian workers who were sampled in the TPHS (*p* = 0.013) and Australian workers in the NSMHWB survey (*p* = 0.002). Prevalence estimates of high psychological distress for women in the population surveys were not statistically different (*p* > 0.05) to those obtained by the researcher survey but they had higher average percentages than those calculated for men.

Within the 16 to 24 year age-group in men the differences in the TSS surveys are also pronounced (18.7% [ref] for the researcher survey and 23.7% [p = 0.002] for the employer survey) when compared with both the TPHS (7.1%, p = 0.132) and the NSMHWB (6.2%, p < 0.001). Prevalence percentages for the employer survey were elevated across all male age-groups. For the TSS surveys, the prevalence of high psychological distress in the 16 to 24 year and 25 to 34 year women’s age-groups also appeared higher than in the population surveys, with these elevations being consistent for all age-groups in the employer survey.

### Correlates of high psychological distress in the public sector workforce

#### *Sample characteristics*

For the researcher survey, the average age of respondents was 45 years and 67% were female (see Supplementary File Table 1A, which shows respondent characteristics for the Partnering Healthy@Work 2010 survey). Most respondents were married (77%), had received education up to Year 10 (54%), were permanently employed (90%), full-time (61%) employees working regular schedules (81%), Monday to Friday (57%). On average, respondents had public sector tenure of 12 years, worked 38 hours a week and received an annual salary of A$66,236. The weighting process showed that the respondents were more likely than non-respondents to be older, female, of longer tenure, full-time employees, and to have worked within specific government departments.

#### Univariable correlates of high psychological distress

Men and women showed many similar univariable correlates for high psychological distress that were statistically significant (p < 0.05) (Table 3). Using the parameters described in the Methods section, marital status, state service tenure, health behaviours, preferred hours and ERI were selected for subsequent model building across both sexes. Although age was only significantly associated with distress for women, it was included in the men’s model due to its clinical importance. For men, a unique association was apparent for employment category and employment condition. Occupation, annual salary and department tenure were uniquely associated with high psychological distress among women. Furthermore, women had a significant association for both ‘less hours’ and ‘more hours’ in the preferred hours category whereas men only met the inclusion criterion for the ‘less hours’ category. For men, state service tenure was added at the conclusion of preliminary main effects modelling [34].

**Table 3:** Univariable logistic regression analysis

	** Men**	*** p***	** Women**	*** p***
***Variables***	** PR**^**1 **^**(95% CI)**		** PR (95% CI)**	
Age (years) continuous	0.99 (0.98 - 1.01)	0.355	0.97 (0.97 – 0.98)	<0.001
16-24	1.00	-	1.00	-
25-34	0.40 (0.12 – 1.35)	0.139	0.98 (.053 – 1.83)	0.959
35-44	0.63 (0.21 – 1.87)	0.406	0.79 (0.43 – 1.45)	0.446
45-54	0.62 (0.21 – 1.78)	0.372	0.62 (0.34 – 1.13)	0.116
55-64	0.38 (0.12 – 1.18)	0.095	0.40 (0.21 – 0.78)	0.007
65+	Insufficient cases	0.000	0.23 (0.03 – 1.70)	0.150
Marital Status				
Not married	2.00 (1.32 - 3.05)	0.001	1.41 (1.10 – 1.79)	.006
Married/Defacto	1.00	-	1.00	-
Occupation				
Blue Collar	1.15 (0.62 -2.13)	0.668	1.24 (0.88 – 1.74)	0.227
Admin	0.94 (0.54 – 1.62)	0.815	1.41 (1.05 – 1.91)	0.023
Service	1.00	-	1.00	-
Professional	1.55 (0.76 – 3.13)	0.228	1.29 (0.60 – 2.80)	0.517
Manager	0.85 (0.49 – 1.46)	0.553	1.35 (0.92 – 1.96)	0.123
Annual Salary	1.01 (0.97 – 1.06)	0.638	0.94 (0.88 – 1.00)	0.044
Education				
Up to Year 10	0.63 (0.26 – 1.53)	0.308	0.92 (0.65 – 1.31)	0.653
Up to Year 12/certificate	1.22 (0.82 – 1.83)	0.322	1.02 (0.80 – 1.33)	0.826
Post School	1.00	-	1.00	-
Job Classification				
Bands 1-3	0.96 (0.58 – 1.59)	0.872	0.92 (0.72 – 1.17)	0.484
Bands 4-6	0.97 (0.51 – 1.85)	0.930	0.86 (0.55 – 1.36)	0.518
Bands 7-8	1.32 (0.64 – 2.72)	0.444	0.56 (0.21 – 1.48)	0.244
Bands 9-10	1.00	-	1.00	-
Employment Category				
Fixed Term/ Casual	1.77 (1.10 – 2.86)	0.018	1.18 (0.79 – 1.77)	0.427
Permanent	1.00	-	1.00	-
Employment Condition				
Part Time	1.41 (0.88 – 2.26)	.0156	0.90 (0.72 – 1.13)	0.351
Full Time	1.00	-	1.00	-
Tenure				
State Service	0.99 (0.97 – 1.00)	0.119	0.98 (0.97 - 0.99)	<0.001
Agency	0.99 (0.97 – 1.01)	0.285	0.98 (0.96 – 1.00)	0.051
Days worked				
Days Vary Weekly	0.83 (0.47 – 1.48)	0.534	1.12 (0.82 – 1.53)	0.463
Other	1.48 (0.89 – 2.44)	0.129	0.85 (0.65 – 1.11)	0.235
Mon to Fri	1.00	-	1.00	-
Schedule worked				
Regular day	1.06 (0.65 – 1.72)	0.812	0.89 (0.66 – 1.19)	0.421
Other types	1.00	-	1.00	-
Hours worked	1.00 (0.98 – 1.02)	0.881	1.00 (1.00 – 1.01)	0.422
Preferred hours				
Less hours	1.49 (1.00 – 2.22)	.052	1.39 (1.10 – 1.76)	0.006
Same hours	1.00	-	1.00	-
More hours	1.26 (0.48 – 3.32)	0.635	1.56 (1.01 – 2.42)	0.047
General Health^2^	1.01 (0.97 - 1.06)	0.661	0.99 (0.98 – 1.01)	0.489
BMI (per unit)	1.05 (1.01 - 1.08)	0.010	1.02 (1.00 – 1.05)	0.038
Alcohol Intake				
High Risk	1.60 (1.05 - 2.44)	0.030	1.61 (1.21 – 2.13)	0.001
Low Risk	1.00	-	1.00	-
Fruit & Veg Intake				
Inadequate	1.41 (0.96 - 2.08)	0.079	1.46 (1.16 – 1.83)	0.001
Adequate	1.00	-	1.00	-
Smoker				
Daily Smoker	1.89 (0.99 - 3.61)	0.053	1.36 (0.94 – 1.97)	0.105
All Others	1.00	-	1.00	-
Physical Activity^3^				
Lower	1.30 (0.78 - 2.18)	0.318	0.89 (0.66 – 1.20)	0.442
Middle	1.92 (1.18 - 3.11)	0.008	1.23 (0.94 – 1.61)	0.134
Upper	1.00		1.00	-
ERI tertiles^4^				
Lower	1.00	-	1.00	-
Middle	1.02 (0.53 – 1.96)	0.950	1.53 (1.00 – 2.35)	0.051
Upper	3.54 (2.10 - 5.97)	<0.001	4.59 (3.20 – 6.60)	<0.001

High psychological distress prevalence ratios for men and women participating in the Partnering Healthy@Work 2010 survey.

^**1**^PR, Prevalence Ratio using inverse probability weighting method.

^2^General Health is derived from SF12 aggregate physical score.

^3^Physical activity tertile ranges (minutes per week) 0-146, 147-252, 253-362 – a combination of total work and leisure minutes.

^4^ERI, Effort-Reward Imbalance tertile ranges: Males = 0.21-0.36 (lower); 0.36 - 0.49 (middle); 0.49 – 2.32 (upper). Females = 0.21 - 0.36 (lower); 0.36 - 0.50 (middle); 0.50 – 2.05 (upper).

The variables contained in the fully adjusted model for men (Table 4) were classified as acceptably discriminating (ROC = 0.77) were age, marital status, employment category, state service tenure, BMI, alcohol use, daily smoking, and perceptions of high-effort and low-reward. This set of variables provided the best discrimination between high and low/moderate psychological distress (ROC = .76), with independent associations being found for younger age, fixed-term/casual employment and the upper tertile of ERI. Weighted population estimates of high distress prevalence by ERI tertiles for men were 5.8% (lower), 5.3% (middle) and 20.5% (upper) respectively. A 3-fold increase in prevalence of psychological distress was evident between the highest and lowest ERI categories in the resultant model.

**Table 4:** Sequential log binomial regression models

	**Men**			**Women**		
***Variables***	**PR**^**1**^	**95% CI**^**2**^	***p***	**PR**	**95% CI**	***p***
Demographic variables						
Age	0.97	0.94 – 1.00	0.016	0.97	0.95 – 0.99	0.007
Marital status	1.31	0.70 – 2.48	0.388	1.44	1.01 – 2.05	0.048
Job characteristics						
Employment category	2.61	1.25 – 5.47	0.011	**-**	**-**	**-**
State Service tenure	0.98	0.95 – 1.01	0.147	0.99	0.96 – 1.01	0.240
Health-risk behaviours						
BMI	1.03	0.97 – 1.09	0.396	1.02	0.99 – 1.05	0.143
Risky alcohol	1.24	0.68 – 2.26	0.479	1.17	0.79– 1.71	0.438
Daily smoker	1.86	0.89 – 3.92	0.101	1.35	0.88 – 2.09	0.168
Inadequate fruit and vegetable intake	**-**	**-**	**-**	1.27	0.88 – 1.80	0.200
Psychosocial factors						
ERI^2^						
Middle	0.79	0.30 – 2.05	0.624	1.52	0.75 – 3.11	0.246
Upper	3.37	1.52 – 7.47	0.003	5.28	2.91 – 9.75	<0.001
*ROC*^*3*^	.7659			.7559		

Correlates of high psychological distress for men and women participating in the Partnering Healthy@Work 2010 survey.

^1^PR, Prevalence Ratio; CI, Confidence Interval.

^2^ERI, Effort-Reward Imbalance tertiles.

^3^ROC, Area under the Receiver Operating Characteristics curve

- Models for men and women were calculated separately.

- Reference categories: married or defacto, permanent employment, not risky alcohol, not daily smoker, ERI lower tertile. Age, state service tenure and BMI are continuous variables.

- Omitted categories, designated with a ‘-‘ identify that the variable did not make a contribution (p>0.05) to the ROC.

Among women, the set of variables providing the best discrimination between high and low/moderate psychological distress (ROC = 0.75) were age, marital status, state service tenure BMI, alcohol use, daily smoking, fruit and vegetable intake and ERI were included in the model. Independent associations with high distress were found in the fully adjusted model for women who were younger, unmarried, and in the upper tertile of ERI (Table 4). Weighted population estimates of high distress prevalence by ERI tertile for women were 4.7% (lower), 7.2% (middle) and 22.0% (upper) respectively.

Weighted population estimates of high distress prevalence by ERI tertile for women were similar to those for men being 4.7% (lower), 7.2% (middle) and 22.0% (upper) respectively. A 5-fold increase in psychological distress prevalence was identified in the highest versus lowest ERI categories.

## Discussion

In this study we compared the self-reported prevalence of high psychological distress between two surveys of a public sector workforce and then with population normative worker surveys using the same K10 brief screening measure. We examined whether there were differences in the results obtained from employer and researcher approaches to data collection and we also investigated the socio-demographic, health and work correlates of high psychological distress

For both sexes, we found that the prevalence of high psychological distress was greater in the employer survey when it was referenced to the researcher survey. This higher prevalence was observed across all age-groups for the employer survey. When the researcher survey was compared with population norms for workers, prevalence of high distress was greater for men but not women. It is interesting that the women’s researcher and population surveys showed strong consistency in prevalence estimates despite their differing modes of data collection (telephone, paper-pencil and interview) and arguably dissimilar workforces.

Age-group differences in prevalence were also identified when comparing the public sector and population surveys, particularly with the youngest male and female age-groups. Psychological distress has been found to be more prevalent among younger people and tends to decrease as age increases [24]. Jorm and colleagues [36] found that problems such as job threats, personal problems and ending relationships were most prominent among younger age groups. Moreover, early working life coincides with a potentially stressful major developmental stage as adolescents move into adulthood and have to cope with associated adult responsibilities [37]. Recent papers have advocated the need to generally target younger workers with mental health promotion strategies [38,39] so although the age-group differences evident in this study are not conclusive, our results are in a direction that is consistent with other research.

Despite previous validation of the online K10 as a screener for depression [40], in this study administration of the K10 by an employer to a volunteer sample of employees appears to have overestimated prevalence. Screening is an important component of prevention and treatment efforts [41] but guidance on interpretation is needed to prevent inaccurate conclusions about the prevalence of distress or where preventive efforts should be targeted. Furthermore, as has been found elsewhere [3], age and sex data make a relatively limited contribution by themselves to our understanding of the drivers of worker psychological distress within organisations. It was a necessary step in this research setting to assess whether high distress, as measured by the K10, was associated with expected correlates. Modelling was necessary to investigate this issue.

The logistic modelling results showed that the best set of variables to predict high psychological distress in both men and women were age, marital status, state service tenure, BMI, alcohol use, daily smoking and ERI, although not all included variables were independently associated with distress. For men, employment category (permanent vs. fixed-term/ casual) was also important, while for women fruit and vegetable intake contributed to model discrimination but was not itself independently associated with distress.

In the men’s fully adjusted model, high psychological distress was independently associated with lower age, fixed term or casual employment and the upper tertile of ERI. High distress prevalence was twice as likely for men in fixed term or casual employment and three times more likely if they were experiencing high effort reward imbalance. It has historically been argued that rationalization of the public sector has contributed to higher levels of psychological distress [9]. Although this public service had not commenced rationalization at the time of the surveys, lag effects of historical organisational changes could be considered. Men have also been found to be at higher risk of psychological distress in the face of job insecurity [42] which can be assessed through employment category and ERI variables. Nevertheless, it is also possible that workers with poorer mental health are more likely to hold precarious temporary employment [43]. Further investigation is needed to establish the direction of effect and factors influencing men’s mental health in this public service.

For the women’s fully adjusted model, younger age, being unmarried, and high ERI were independently associated with high psychological distress, with ERI making the major contribution. The results estimate that the prevalence of distress among those in the upper tertile of ERI was 20% for men and 22% for women. This finding, combined with longitudinal evidence of association [44], suggests that if attention is given to addressing the risk of effort-reward imbalance, there may be a related reduction in the risk of high psychological distress in the workplace. The reasons for the strength of the ERI contribution to the models are an area for further investigation. As an example, Tsutsumi and Kawakami [45] have proposed that extrinsic efforts (e.g. overtime, workload) and rewards (e.g. praise, development opportunities) can be a focus of organisational interventions to overcome imbalance. Few interventions have yet been developed based on improving ERI but early attempts show promise (e.g. [44]).

Overall, the models presented here suggest priorities for mental health intervention would include men in fixed-term or casual employment, younger workers and those experiencing high Effort-Reward Imbalance. Responses based on randomly sampled, weighted data identified correlates of high psychological distress that are reasonably consistent with other international studies, and provided direction as to sub-populations needing targeting through workplace mental health promotion strategies. These findings provide support for use of the K10 as a brief screening measure of psychological distress within an organisational context, providing appropriate guidance on interpretation is available.

### Limitations

These study implications need to be examined in view of several limitations. First, participation rates were below 30% for both of the public sector surveys, though these rates are typical for the field [3]. A key strength of this study is the use of weighting procedures to help control for response bias in the researcher survey. In comparison, the employer survey was unweighted and may not have provided a reliable indication of psychological distress prevalence in this public sector workforce. Second, it is possible that the differences in survey data collection methods are biasing the results. However, Mealing and colleagues have argued against this position [46], finding that relative risk estimates were consistent for studies of the same population with different methods of data collection, response rates and sampling methods as long as survey questions were the same. A recent study has also found no significant differences between response rates, or types of responses to questions whether surveys were perceived to be anonymous or not [47]. We acknowledge that the population studies we have used as comparators may have underestimated the prevalence of psychological distress due to non-response bias [18]. However, we required points of adjudication because the workforce survey results were conflicting and these norms were the best estimates available. Third, this study is cross-sectional and the direction of relationships cannot be established. Kolstadt and coworkers [48] have also suggested that reporting bias can inflate associations between self-reported job-strain and depression, and a work unit analysis may show weaker associations than those found here. Finally, there is room for improvement in the types of measures applied in this study. For instance, our study used the socioeconomic variable ‘annual salary’ as a proxy measure for income, which may be an underestimate for occupations with allowances and overtime (e.g. police, nurses). Future measurement of additional contextual measures also seems likely to strengthen our understanding of the variables discriminating high from low psychological distress in public sector workforces.

### Implications for practice: how should these findings be used?

After the employer survey was conducted, this public sector organisation acted on the findings by implementing a mental health promotion strategy that was predominantly focused upon general education about mental health. The impact of this approach is yet to be determined as follow-up data collection is underway, but this study suggests a greater reduction in risk may be obtained if specific sub-populations with poor mental health (i.e. men and younger workers) and people with high effort-reward imbalance are targeted. Anonymous, volunteer surveys of worker mental health may be resource efficient and promote participation but this example suggests they may not advance management decision-making. Health Risk Appraisals for risk reduction rely on valid assessments of health outcomes. Inaccurate results could lead to expensive resource allocation that is ineffective in reducing the risk of poor mental health in the workplace. In addition, mental health screening may also only be of limited use without the collection of at least some of the known important correlates (e.g. individual characteristics, psychosocial factors) to allow specific targeting of interventions. Clinicians have an important role in educating employers about mental health screening and giving advice on health survey interpretation.

## Conclusions

Understanding the nature of workplace-based risks associated with poor mental health and addressing these risk factors is a pressing public health issue. Depression and anxiety screening forms part of a response to this challenge but this study showed that screening using typical employer survey methods with a validated measure could overestimate prevalence. Depression and anxiety screening should be promoted to employers and employees but guidance is needed on interpretation. In identifying priority groups for intervention, perceived work stress and a fixed term/casual employment contract may be particularly important among men.

## Abbreviations

K10: Kessler-10; ERI: Effort reward imbalance.

## Competing interests

The authors declare they have no competing interests.

## Authors’ contributions

This work was competed as part of LJ’s PhD, supervised by KS, AV and AM. LJ and KS planned the design of the manuscript, KS, AV, AM, BT and RT negotiated with the organisations and organised the data collection, LJ and PO analysed the data, LJ wrote the first draft of the manuscript and KS, AV, A M, RT and BT critically commented it and participated in the editing of the final version. All authors have approved the final version of the manuscript.

## Pre-publication history

The pre-publication history for this paper can be accessed here:

http://www.biomedcentral.com/1471-2458/14/125/prepub
